# The Extent of Illicit Cigarette Sales in Five Rural Districts of Pakistan: A Cross-sectional Study

**DOI:** 10.1093/ntr/ntae155

**Published:** 2024-07-15

**Authors:** S M Abdullah, Saeed Ansaari, Melanie Boeckmann, Amina Khan, Kamran Siddiqi

**Affiliations:** Department of Health Sciences, University of York, York, UK; Department of Economics, University of Dhaka, Dhaka, Bangladesh; Research and Development, ARK Foundation, Dhaka, Bangladesh; Public Health Department-Research, The Initiative, Islamabad, Pakistan; Department of Global Health, Institute of Public Health and Nursing Research, University of Bremen, Bremen, Germany; Public Health Department-Research, The Initiative, Islamabad, Pakistan; Department of Health Sciences, University of York, York, UK

## Abstract

**Introduction:**

The Illicit Tobacco Trade (ITT) subverts tobacco control efforts. Cigarette packs sold without legal health warnings undermine efforts to warn the public about the dangers of tobacco. Furthermore, cigarettes sold below minimum retail prices are indicative of tax evasion leading to revenue loss and budgetary deficits in high tobacco-burden economies. The extent of the ITT in rural areas of such countries might differ from urban. We estimated the extent of illicit cigarette sales in selected rural areas of Pakistan.

**Aims and Methods:**

We analyzed cigarette packs collected from 85 villages in Pakistan as part of a cross-sectional consumer survey of 2550 rural households. We classified cigarette packs as noncompliant if these were missing: A text health warning, pictorial health warning (PHW), underage sale prohibition warning, retail price, or manufacturer details. To measure the extent of tax evasion, we estimated the proportion of packs purchased below the legal minimum retail price.

**Results:**

Only 35% (429/1228) of rural smokers were able to show their cigarette packs. Out of these, 89% (382/429) of packs were noncompliant with the cigarette packaging and labeling laws. In rural areas, 83% (357/429) of packs did not have PHW and 33.8% (145/429) did not have printed retail prices. Among all packs, 41% (177/429) were purchased below the minimum retail price of 63 Pakistani Rupees and hence highly likely to have evaded taxes.

**Conclusions:**

We found a very high previously unreported proportion of noncompliant cigarette packs in selected rural areas of Pakistan indicating weaker implementation of tobacco control laws in rural areas.

**Implications:**

This paper presents previously unreported estimates of the share of illicit cigarette sales in rural areas of Pakistan. Most packs (89%) in our sample were noncompliant with the packaging and labeling regulations and a significant proportion (41%) were purchased below the minimum price. The extent of illicit tobacco was found to be far greater in rural than in urban areas of Pakistan. Taking advantage of poor law enforcement, the tobacco industry may be complicit in flooding the rural markets with illegal and cheap cigarettes. Given this disparity, law enforcement authorities must focus on rural areas.

## Introduction

With a global prevalence of 22.3%, tobacco is considered a major public health concern causing more than 8 million deaths every year.^1^ Illicit Tobacco Trade (ITT) continues to contribute to this public health threat. Eliminating ITT may reduce tobacco demand and increase government revenue.^2^ However, ITT, owing to its diverse and complex nature, in many instances remains beyond the scope of routine practices for tobacco control and can undermine policy effects. A substantial policy design concern is estimating the share of ITT in varying contexts and specifying potential drivers.

Globally, the illicit cigarette share has remained stable in the past decade (11.6% in 2007 and 11.2% in 2018).^2–4^ However, many of these estimates are based on studies in high-income countries.^5^ Evidence on the extent to which ITT features in rural areas of low- and middle-income countries with high tobacco burden is scarce. The extended distribution of illicit cigarettes and more importantly, the rural dynamics for the problem are seldom explored.

Pakistan—a high tobacco burden country (overall tobacco prevalence 19.1%)—has nearly 65% of the population living in rural areas.^6–8^ The rural tobacco use prevalence is 21.1% which is 5.2 percentage points higher than that in urban (15.9%).^7,9^ For different types of tobacco; the difference between rural and urban prevalence is almost four percentage points for smoking (13.9% vs. 10.0%) and 1.5 percentage points (8.2% vs. 6.7%) for smokeless tobacco. The daily average smoking intensity in urban and rural areas is 14.1 and 13.3 sticks, respectively. Nevertheless, rural smokers initiate at a relatively younger age (mean 18.4 years) than their urban counterparts (mean 19.5 years). Passive smoking exposure at home is 55.7% in rural areas as opposed to 36.7% in urban.^7,9^ Among annual 161 000 tobacco-attributable deaths in Pakistan, 31 000 are due to exposure to secondhand smoke.^10^ The greater exposure to passive smoking in rural areas can result in a disproportionately larger tobacco-attributable disease burden. Illicit tobacco sales in rural areas hence have significant adverse health implications.

Urban estimates on ITT cannot be generalized to rural areas due to differences in the context and composition of tobacco users and tobacco markets. Due to often lower socioeconomic profiles, rural dwellers demand lower-priced tobacco than their urban counterparts.^11,12^ Tobacco retailers in rural areas might be driven to meet this demand with low-price tobacco, which in turn is often illicit. ITT in rural areas may also run a lower risk of being inspected and fined by the authorities than in urban areas due to stricter law enforcement in cities. On the other hand, affluent urban residents may often demand imported cigarette brands which are of high price and may be smuggled in. Nevertheless, tobacco control efforts are generally more intense in urban than rural areas.^13^ Owing to this, rural areas may be low-risk alternative destinations for illegal practices related to tobacco sales.

Research on ITT estimates in low- and middle-income countries, particularly in rural areas, is scarce.^14–17^ For Pakistan, the estimates of illicit cigarette sales in cities have been published elsewhere.^6^ This report focuses on illicit cigarette sales in selected rural areas of Pakistan.

## Materials and Methods

In phase II of a nationwide cross-sectional STOP (Studying Tobacco Users of Pakistan) survey, we collected cigarette packs from smokers living in the rural areas of Pakistan and analyzed those to estimate the proportion of illicit cigarette sales. In phase I, we focused on the urban population; those methods and findings are published elsewhere.^6,18^

This was a face-to-face household survey of tobacco users conducted between December 2021 and March 2022. According to Census 2017, we selected the top two districts (Faisalabad and Muzaffargarh) in Punjab with the highest rural population and one district each from the other three provinces (Khairpur in Sindh, Peshawar in Khyber Pakhtunkhwa and Quetta in Baluchistan) also based on the highest rural population, respectively. Altogether, these districts represent 14% (18 million/132 million) of Pakistan’s rural population. Eligible households, with at least one tobacco user aged 15 and above, were selected using a two-stage random sampling method. At first, 85 villages (primary sampling units) were randomly selected from the five districts, using the probability proportional to size method -number of households as a measure of size. In each primary sampling units, from the set of eligible households, 30 were selected randomly for the survey resulting in a total sample size of 2550 households. The sample size was based on assuming a smoking prevalence of 13.9% and a design effect equal to 2 in rural areas according to the latest Global Adult Tobacco Survey.^7^ Based on the latest population census (2017),^8^ the population at risk and the average household size were assumed to be 55.1% and 6.6%, respectively. The response rate was set to 95% and a margin of error of 0.075.

One tobacco user per household was recruited using the Kish Grid method.^19^ Before recruitment, potential participants received written and verbal information and those interested signed written consent. Among those recruited (2550), cigarette smokers (1228) were asked to share their cigarette packs with the enumerator. The packs were returned to the consumers after taking a six-sided photograph. In addition, data were also collected on their sociodemographics, nicotine dependence (for Heaviness of Smoking Index [HSI])^6,20^ and the purchase price paid for packs. All personally identifiable information was removed from the questionnaire and database.

The illicit cigarette packs were grouped into two categories: Noncompliant and tax-evaded packs. Packs were considered noncompliant if they did not comply with the packaging and labeling regulations^21,22^: presence of a text health warning (text health warning in Urdu on the front and in English on the back of a cigarette pack), pictorial health warning (PHW; PHW size at least 60% and placed on the top on both sides of the pack), underage sale prohibition warning label (sale to under 18 is prohibited by law), and printed retail price (minimum retail price 63 PKR) and manufacturer’s details. The above criteria were based on Pakistan’s existing tobacco control laws and applied by other illicit tobacco research in the country.^6^ The estimates were stratified by sociodemographics and HSI of rural smokers to understand the consumption pattern.^6^

In the absence of a mandatory fiscal marker on the cigarette packs (which only came into effect in Pakistan in July 2022), we used the “price threshold method” to determine the likelihood of tax evasion.^16,23,24^ According to this method, a cigarette pack is likely to have evaded tax if its purchase price is lower than the legal minimum price.^23,24^ Furthermore, noncompliant packs were cross-tabulated against their purchase price with a threshold value of 63 PKR (legal minimum price) to estimate the share of noncompliant and tax-evaded packs.^6^ All estimates were calculated with a 95% confidence interval. STATA V.17.0 was used for statistical analysis.^25^

## Results

A total of 1228 smokers were surveyed from the selected rural areas of Pakistan covering all provinces. Among these, 26.4% (95% CI: 24.0 to 29.1) smokers reported that they bought only loose cigarettes, 35.7% (95% CI: 33.3 to 38.8) discarded their packs, and another 3% (95% CI: 2.1 to 4.1) borrowed cigarettes from others. Hence, 34.9% (95% CI: 32.2 to 37.5) of the smokers (*n* = 429) were able to show their packs for further examination (Table S1A).

**Figure 1.**
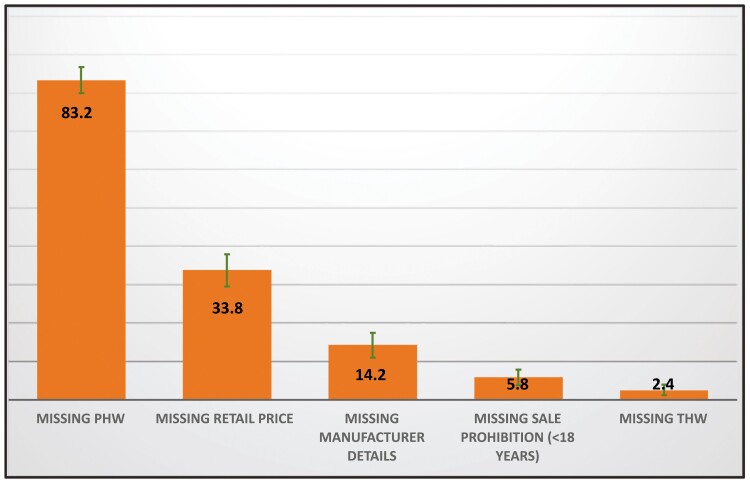
Distribution of cigarette packs against compliance criteria in rural Pakistan

Based on national tobacco control laws in Pakistan, the collected cigarette packs were examined for compliance using five criteria. Figure 1 provides the share of noncompliant packs for each criterion. Among 429 cigarette packs, 382 (89%; 95% CI: 85.8 to 91.8) were noncompliant with at least one of the five criteria. The most common noncompliant feature was missing PHW (not of sufficient size in proportion or not in the right place; 83%; 95% CI: 80.0 to 86.7).

We found that the top five cigarette brands in rural Pakistan were Capstan (*n* = 148 [34.5%]; noncompliant = 116 [30.4%]), Morven (*n* = 58 [13.5%]; noncompliant = 54 [14.1%]), Kissan (*n* = 55 [12.8%]; noncompliant = 55 [144%]), Gold Flake (*n* = 40 [9.3%]; noncompliant = 39; [10.2%]) and Champion (*n* = 15 [3.4%]; noncompliant = 15; [3.9%]). Most Gold Flake (92.5%) and Morven cigarette (84.5%) packs had missing PHW (Table S2A). Out of the total 429 packs collected from rural smokers, 181 packs (33.8%; 95% CI: 29.8 to 38.2) either did not have printed retail price or the printed price was below the legislated minimum price (63 PKR; Table 1).

**Table 1.** Practice Related to Legislated Minimum Cigarette Price (63 PKR)—Indication for Tax Evasion

Category	Total packs (*N* = 429)			Total noncompliant packs (*N* = 382)		
	*n*	%	95% CI	*n*	%	95% CI
No retail price printed or printed price < 63 PKR	181	42.2	[37.5 to 47.0]	169	44.3	[39.2 to 49.4]
Purchase price < 63 PKR	177	41.3	[36.6 to 45.9]	166	43.0	[38.1 to 48.3]
Printed retail price < 63 PKR & purchase pricE < 63 PKR	33	7.7	[5.4 to 10.5]	23	6.0	[3.4 to 8.1]

Table S1A contains the distribution of packs against their noncompliant status stratified by sociodemographics. The consumption of noncompliant and compliant cigarettes has similar patterns over the age group and educational attainment of smokers. Nevertheless, A higher proportion of smokers consuming noncompliant cigarette packs were in the 51–65 age bracket (30%; 95% CI: 26 to 35) as compared to those using compliant packs (19%; 95% CI: 9 to 30); however, the difference was nonsignificant. Half of the noncompliant packs in the selected rural areas (50%; 95% CI: 44.8 to 55.0) were consumed by smokers with no formal education. Rural smokers with low nicotine dependence (measured with low HSI) consume more noncompliant cigarettes (55%; 95% CI: 49.9 to 59.8) than those with high nicotine dependency.

In total, 177 out of 429 packs (41.3%; 95% CI: 36.6 to 45.9) were purchased below the minimum price. Among those considered noncompliant, 166 out of 382 packs (43%; 95% CI: 38.1 to 48.3) were purchased below the minimum price. All noncompliant Kissan and Champion cigarette packs were purchased for a price less than the legal minimum price of 63 PKR. All packs purchased below the minimum retail price were considered as most likely to have evaded tax.

We also compared the characteristics of smokers who showed their cigarette packs with those who did not (Table S3A). The age distribution, geographical spread, and level of education were similar in both groups; however, the group that showed cigarette packs were more nicotine dependent than the group that did not (9% [95% CI: 6.3 to 12] vs. 3% [95% CI: 1.8 to 4.4] in the high HSI category).

## Discussion

Cigarette packs that did not comply with the packaging and labeling regulations were found to be highly prevalent (89%) in selected rural areas of Pakistan. Most of the packs (83%) were noncompliant with PHW. We observed that a large proportion of all (41%) and noncompliant cigarette packs (43%) were sold below the legal minimum price. These are important findings as cigarette packs sold without legal health warnings undermine efforts to warn the public about the dangers of tobacco. Furthermore, cigarettes sold below minimum retail prices are indicative of tax evasion leading to revenue loss and budgetary deficits in high tobacco-burden economies. The tobacco industry is likely to be complicit in flooding the rural markets in Pakistan with noncompliant cigarette packs. In the absence of a functioning track and trace system in Pakistan at the time of this research, tax collection relied on the tobacco industry reporting to the revenue collectors. A high proportion of cigarette packs sold below the minimum retail price could mean that the industry failed to report these packs and evaded taxes.

In the selected rural areas, consumption of noncompliant cigarettes varied with educational attainment with 50% of those being smoked by uneducated smokers. PHW is an effective tool for communicating the risks of smoking.^26,27^ Quit intentions and attempts are more likely to be among smokers with exposure to PHW.^27,28^ Alongside the public health perspective, such a high prevalence of noncompliant cigarettes has economic implications.

There is a large urban–rural disparity in tobacco control activities in Pakistan: the proportion of noncompliant packs as well as those with a high likelihood of tax evasion in selected rural areas, was found to be higher than in urban areas in our survey. In total, 17.8% (89%) of all the packs collected from urban (rural) smokers were noncompliant and 13.8% (41.3%) sold below the legislated minimum price.^6^ Though limited, such disparity in illicit proportion with a rural predominance is also evident in other low- and middle-income countries (eg, Turkey; Rural—15.9% vs. Urban—9.8%, Brazil; Rural—53.6% vs. Urban—28.6%).^14,16^ Rural areas are naturally isolated and the intensity of tobacco control efforts remains less than in urban.^13,29^ The relatively high prevalence of noncompliant and tax-evaded cigarette sales in rural areas, in addition to the high tobacco burden (measured with higher prevalence, higher smoking intensity, and higher exposure to passive smoking) indicates the implementation weakness of tobacco control laws in those areas in Pakistan.

The high prevalence of loose selling of cigarettes poses a challenge to tobacco control in the country.^30^ Although banned, we found that in selected rural areas in Pakistan, the prevalence of loose purchases was 26.4% (95% CI: 24.0 to 29.1). Similar to the urban distribution, in the selected rural areas we found relatively younger smokers with low educational attainment and low nicotine dependence purchase loose cigarettes.^6^

The noncompliant cigarette brands in the rural areas also differed from those found in the urban areas. There were more domestic brands: Pakistan Tobacco Company (Capstan and Gold Flake), Philip Morris International (Morven), Royal Tobacco Company (Champion), and Khyber Tobacco Company (Kissan). In the urban survey, only Gold Flake and Kissan were spotted in the top five list of noncompliant packs.^6^ Furthermore, the five most common brands found in rural areas were also mentioned among the most popular rural brands in a recent report published by the Social Policy and Development Center.^31^ Effective tobacco regulation with geographically equitable implementation is thus necessary to control the high prevalence of noncompliant and tax-evaded cigarette sales and hence overall tobacco.

The study has a few limitations. Only one-third of the surveyed smokers showed their cigarette packs (429 packs from 1228 rural smokers). The estimated share of noncompliant packs might be underestimated if the smokers who were reluctant to show packs had been carrying noncompliant packs. Nevertheless, the scope of such underestimation is limited as already 89% of the examined packs were noncompliant. Although the number of packs was low and hence representativeness could be a concern, the sample had smokers from all the provinces of Pakistan. Three districts (Peshawar, Quetta, and Faisalabad) included either the provincial capitals or major cities and therefore might not represent remote areas. Nevertheless, given the general lack of enforcement of tobacco control laws in Pakistan, it is still plausible that the rural areas may not get the same level of inspections as major cities despite being close to them. Given the absence of a gold standard (eg, “excise stamp authenticity”) for measuring the proportion of tax-evaded cigarettes, we used the “price threshold method” and compared printed and purchased prices of cigarettes with the legislated minimum price. It is implausible that those who have not printed the price or printed it below the minimum price, or sold it below the minimum price have paid the legal tax for cigarettes. The rural survey took place a year after the urban survey, hence interim policy changes could not be controlled for. Moreover, differentiating counterfeit cigarettes was out of the scope of work.

Tackling noncompliance in cigarette packs and tax evasion in rural areas would require a proper track and trace system. Since July 2022, affixing tax stamps on cigarette packs has been made obligatory in Pakistan.^32^ To ensure adherence, tobacco control law enforcement in rural areas needs strengthening. Future research analyzing the cigarette tax stamps using our methods can help to understand the trajectory of tax evasion under the recently implemented track and trace system.

## Supplementary material

Supplementary material is available at *Nicotine and Tobacco Research* online.

ntae155_suppl_Supplementary_Tables

## Data Availability

Data are available on reasonable request.
