# Supplementary material for: The Extent of Illicit Cigarette Sales in Five Rural Districts of Pakistan: A Cross-sectional Study
Source: Nicotine Tob Res. 2024 Jul 15;27(1):143–7. doi: 10.1093/ntr/ntae155 (PMC11663807; doi:10.1093/ntr/ntae155)
Supplement: ntae155_suppl_Supplementary_Tables [file ntae155_suppl_supplementary_tables.pdf]

Supplementary

Table 1A: Collected cigarette packs and their distributions against non-compliant status and smokers' characteristics

Distribution of 1228 rural smokers against the pack availability during survey			
**Pack Availability**	**Frequency**	**Percentage (%)**	**CI (95%)**
Pack Shown	429	34.9	[32.2-37.5]
Pack Discarded	438	35.7	[33.3-38.8]
Borrowed Cigarette	37	3.0	[2.1-4.1]
Bought Loose	324	26.4	[24.0-29.1]

Extended distribution of 429 packs showed by the rural smokers against their illicit status							
**Smokers Characteristcs**	**Category**	**Non-compliant Packs (N=382)**			**Licit Packs (N=47)**		
		**Packs (n)**	**%**	**CI (95%)**	**Packs (n)**	**%**	**CI (95%)**
Age	15-30	33	8.6	[5.8-11.8]	8	17.0	[6.4-29.8]
	31-50	206	53.9	[48.7-59.2]	29	61.7	[48.9-76.6]
	51-65	116	30.4	[25.7-35.1]	9	19.1	[8.5-29.8]
	>65	27	7.1	[4.5-9.7]	1	2.1	[0.0-6.4]
Gender	Male	380	99.5	[98.7-100]	47	100.0	[100-100]
	Female	2	0.5	[0.0-1.3]	-	-	-
Marital Status	Single	21	5.5	[3.4-7.9]	4	8.5	[2.1-17]
	Engaged	1	0.3	[0.0-0.8]	42	89.4	[80.9-97.9]
	Married	350	91.6	[88.7-94.2]	1	2.1	[0.0-6.4]
	Separated	-	-	-	-	-	-
	Divorced	-	-	-	-	-	-
	Widow	10	2.6	[1.3-4.5]	-	-	-
Education	None	191	50.0	[44.8-55]	27	57.4	[42.6-72.3]
	<Primary	32	8.4	[5.8-11.3]	5	10.6	[2.1-21.3]
	Primary	48	12.6	[9.2-16.5]	2	4.3	[0.0-10.6]
	<Secondary	40	10.5	[7.3-13.9]	6	12.8	[4.3-23.4]
	Secondary	29	7.6	[5.0-10.5]	3	6.4	[0.0-14.9]
	High School	33	8.6	[5.8-11.5]	3	6.4	[0.0-14.9]
	Graduate	9	2.4	[1.0-3.9]	1	2.1	[0.0-6.4]
	Postgraduate	-	-	-	-	-	-
Heaviness of Smoking Index (HSI)	Low	204	55.00	[49.9-59.8]	16	34.8	[21.7-50]
	Moderate	139	37.50	[32.6-42]	21	45.7	[30.4-58.7]
	High	28	7.50	[5.1-10.5]	9	19.6	[8.7-30.4]

Table 2A: Brand-wise distribution of cigarette packs with the non-compliant status

Criteria	Capstan (N=148)			Morven (N=58)			Kissan (N=55)			Gold Flake (N=40)			Champion (N=15)			Others (N=113)		
	n	%	CI (95%)	n	%	CI (95%)	n	%	CI (95%)	n	%	CI (95%)	n	%	CI (95%)	n	%	CI (95%)
Missing/Improper THW	-	-	-	-	-	-	-	-	-	-	-	-	-	-	-	10	8.8	[4.3 - 15.6]
Missing/Improper PHW	114	77	[69.4 - 83.5]	49	84.5	[72.5 - 92.6]	55	100	[93.5 - 100]	37	92.5	[79.6 - 98.4]	8	53.3	[26.5 - 78.7]	94	83.2	[74.9 - 89.5]
Absence of Manufacturer Details	5	3.4	[1.1 - 7.7]	-	-	-	1	1.8	[0.04 - 9.7]	3	7.5	[1.5 - 20.0]	3	20.0	[4.3 - 48.0]	13	11.5	[6.2 - 18.8]
Absence of Sale Prohibition (<18 Years)	6	4.1	[1.5 - 8.6]	2	3.4	[0.4 - 11.9]	-	-	-	1	2.5	[0.06 - 13.1]	3	20.0	[4.3 - 48.0]	13	11.5	[6.2 - 18.8]
No Retail Price Printed or Printed Price<63 PKR	-	-	-	14	24.1	[13.8 - 37.1]	55	100	[93.5 - 100]	2	5	[0.6 - 16.9]	14	93.3	[68.0 - 99.8]	93	82.3	[73.9 - 88.8]
Purchase Price<63 PKR	-	-	-	12	20.7	[11.1 - 33.3]	55	100	[93.5 - 100]	-	-	-	15	100	[78.0 - 100.0]	95	84.1	[75.9 - 90.2]
Non-compliant packs	116	78.4	[70.8 - 84.7]	54	93.1	[83.2 - 98.0]	55	100	[93.5 - 100]	39	97.5	[86.8 - 99.9]	15	100	[78.0 - 100.0]	103	91.2	[84.3 - 95.6]

Table 3A: Distribution of pack showing status over the smokers' characteristics

Smokers Characteristics	Category	Packs Shown (N=429)			Packs Not Shown (N=799)											
					Loose Purchase (N=324)			Discarded Packs (N=438)			Borrowed (N=37)			Total (N=799)		
		n	%	CI (95%)	n	%	CI (95%)	n	%	CI (95%)	n	%	CI (95%)	n	%	CI (95%)
Province	Punjab	273	63.6	[58.8 - 68.1]	193	59.6	[54.0 - 64.9]	287	65.5	[60.8 - 69.97]	11	29.7	[15.8 - 46.9]	491	61.5	[57.9 - 64.8]
	KPK	16	3.7	[2.1 - 5.9]	52	16	[12.2 - 20.5]	64	14.6	[11.4 - 18.2]	-	-	-	116	14.5	[12.1 - 17.1]
	Sindh	139	32.4	[27.9 - 37.0]	38	11.7	[8.4 -15.7]	77	17.6	[14.1 - 21.4]	13	35.1	[20.2 - 52.5]	128	16.0	[13.5 - 18.7]
	Balochistan	1	0.2	[0.0 - 1.2]	41	12.7	[9.2 - 16.7]	10	2.3	[1.1 - 4.1]	13	35.1	[20.2 - 52.5]	64	8.0	[6.2 - 10.1]
Age	15-30	41	9.6	[6.9 - 12.7]	47	14.5	[10.8 - 18.8]	48	11.0	[8.1 - 14.2]	12	32.4	[18.0 - 49.7]	107	13.4	[11.1 - 15.9]
	31-50	235	54.8	[49.9 - 59.5]	171	52.8	[47.1 - 58.3]	262	59.8	[55.0 - 64.4]	16	43.2	[27.0 - 60.5]	449	56.2	[52.6 - 59.6]
	51-65	125	29.1	[24.8 - 33.6]	87	26.9	[22.1 - 32.0]	100	22.8	[18.9 - 27.0]	7	18.9	[7.9 - 35.1]	194	24.3	[21.3 - 27.4]
	>65	28	6.5	[4.3 - 9.2]	19	5.9	[3.5 - 9.0]	28	6.4	[4.2 - 9.1]	2	5.4	[0.06 - 18.1]	49	6.1	[4.5 - 8.0]
Gender	Male	427	99.5	[98.3 - 99.9]	321	99.1	[97.3 - 99.8]	436	99.5	[98.3 - 99.9]	37	100.0	[90.0 - 100]	794	99.4	[98.5 - 99.7]
	Female	2	0.5	[0.05 - 1.6]	3	0.9	[0.01 - 2.6]	2	0.5	[0.05 - 1.6]	-	-	-	5	0.6	[0.2 - 1.4]
Marital Status	Single	25	5.8	[3.8 - 8.4]	11	3.4	[1.7 - 5.9]	15	3.4	[1.9 - 5.5]	7	18.9	[7.9 - 35.1]	33	4.1	[2.8 - 5.7]
	Engaged	1	0.2	[0.0 - 1.2]	15	4.6	[2.6 - 7.5]	5	1.1	[0.3 - 2.6]	3	8.1	[1.7 - 21.9]	23	2.9	[1.8 - 4.2]
	Married	392	91.4	[88.3 - 93.8]	288	88.9	[84.9 - 92.0]	407	92.9	[90.0 - 95.1]	26	70.3	[53.0 - 84.1]	721	90.2	[87.9 - 92.2]
	Separated	-	-	-	1	0.3	[0.0 - 1.7]	1	0.2	[0.005 - 1.2]	-	-	-	2	0.3	[0.03 - 0.09]
	Divorced	1	0.2	[0.0 - 1.2]	-	-	-	2	0.5	[0.05 - 1.6]	-	-	-	2	0.3	[0.03 - 0.09]
	Widow	10	2.3	[1.1 - 4.2]	9	2.8	[1.2 - 5.2]	8	1.8	[0.7 - 3.5]	1	2.7	[0.06 - 14.1]	18	2.3	[1.3 - 3.5]
Education	None	218	50.8	[45.9 - 55.6]	147	45.4	[39.8 - 50.9]	206	47.0	[42.2 - 51.8]	18	48.6	[31.9 - 65.6]	371	46.4	[42.9 - 49.9]
	<Primary	37	8.6	[6.1 - 11.6]	23	7.1	[4.5 - 10.4]	58	13.2	[10.2 - 16.7]	3	8.1	[1.7 - 21.9]	84	10.5	[8.4 - 12.8]
	Primary	50	11.7	[8.7 - 15.0]	31	9.6	[6.5 - 13.3]	72	16.4	[13.0 - 20.2]	1	2.7	[0.06 - 14.1]	104	13.0	[10.7 - 15.5]
	<Secondary	46	10.7	[7.9 - 14.0]	32	9.9	[6.8 - 13.6]	30	6.8	[4.6 - 9.6]	2	5.4	[0.06 - 18.1]	64	8.0	[6.2 - 10.1]
	Secondary	32	7.5	[5.1 - 10.3]	42	13.0	[9.5 - 17.1]	35	8.0	[5.6 - 10.9]	6	16.2	[6.1 - 32.0]	83	10.4	[8.3 - 12.7]
	High School	36	8.4	[5.9 - 11.4]	40	12.3	[8.9 - 16.4]	26	5.9	[3.9 - 8.5]	5	13.5	[4.5 - 28.7]	71	8.9	[7.0 - 1.1]
	Graduate	10	2.3	[1.1 - 4.2]	9	2.8	[1.2 - 5.2]	9	2.1	[0.9 - 3.8]	1	2.7	[0.06 - 14.1]	19	2.4	[1.4 - 3.6]
	Postgraduate	-	-	-	-	-	-	2	0.5	[0.05 - 1.6]	1	2.7	[0.06 - 14.1]	3	0.4	[0.07 - 1.0]
Heaviness of Smoking Index (HSI)	Low	220	52.80	[47.8 - 57.6]	225	78.70	[73.4 - 83.2]	247	58.7	[53.8 - 63.4]	24	77.4	[58.9 - 90.4]	496	67.2	[63.6 - 70.5]
	Moderate	160	38.40	[33.6 - 43.2]	56	19.60	[15.1 - 24.6]	157	37.3	[32.6 - 42.1]	7	22.6	[7.9- 35.1]	220	29.8	[25.5 - 33.2]
	High	37	8.90	[6.3 - 12.0]	5	1.70	[0.5 - 4.0]	17	4.0	[2.3 - 6.3]	-	-	-	22	3.0	[1.8 - 4.4]
